# Surgical Treatments for Legg-Calvé-Perthes Disease: Comprehensive Review

**DOI:** 10.2196/27075

**Published:** 2021-05-03

**Authors:** Arash Maleki, Seyyed Mohammad Qoreishy, Mohammad Nabi Bahrami

**Affiliations:** 1 Orthopedic Department Shahid Beheshti University of Medical Sciences Tehran Iran

**Keywords:** surgical treatment, Legg-Calvé-Perthes disease, pediatric, hip, treatment outcome

## Abstract

**Background:**

Legg-Calvé-Perthes disease (LCPD) is a common public health problem that usually occurs between the ages of 4 and 8 years, but it can occur between the ages of 2 and 15 years. This condition occurs due to the interruption of blood supply to the femoral head. Up to now, different surgical and nonsurgical treatments, including femoral varus osteotomy, innominate osteotomy, pelvic osteotomies, triple osteotomy, Chiari osteotomy, and shelf acetabuloplasty, have been suggested for noncontainable LCPD hips.

**Objective:**

The aim of this comprehensive review was to investigate the various surgical techniques used for LCPD.

**Methods:**

An advanced electronic search of the English-language literature was performed from October 8 to 14, 2020. The electronic databases PubMed, MEDLINE, Web of Science, Embase, Ovid, and Google scholar were searched using appropriate search terms. A manual search of references also was performed. After retrieving the studies, duplicates were removed, and the remining studies were screened based on the title, abstract, and full text. The quality of the selected articles was assessed, and the required data were extracted from eligible articles.

**Results:**

A total of 22 studies were included in the review. Based on the results of the reviewed studies, there are three main factors that influence the treatment outcomes in patients with Perthes disease. These factors are onset age, femoral head involvement severity, and treatment method. The disease has a poor prognosis in children over 8 years old, but this group of patients can also benefit from advanced surgical methods. In patients aged less than 6 years, the disease has a generally good prognosis, but in those aged between 6 and 8 years, its prognosis is variable. Thus, the need for surgical intervention requires close observation of signs. Once any head signs are observed, dynamic arthrography is beneficial before choosing the treatment approach.

**Conclusions:**

This review provides clinicians with a brief guideline for the treatment of patients with LCPD.

## Introduction

Legg-Calvé-Perthes disease (LCPD) is a common childhood disease that commonly occurs between the ages of 4 and 8 years, but it can be found between the ages of 2 and 15 years. This condition occurs owing to the interruption of blood supply to the femoral head. The disease, which is described as aseptic necrosis of the juvenile femoral head, affects about 10 in 100,000 children worldwide [1-3]. Therefore, it is a common condition of the hip in childhood that was first recognized in 1910 by three physicians working independently, including Thornton Legg, Jacques Calvé, and Georg Perthes [4,5]. LCPD is characterized by idiopathic osteonecrosis of the femoral epiphysis that is attributed to arterial infarction [6]. Waldenström has indicated that the process of disease progression commences with aseptic necrosis, followed by a subchondral fracture and fragmentation, revascularization, and remodeling [7,8]. The prevalence of the disease is higher in boys than in girls [9,10]. Additionally, it is more prevalent between the ages of 4 and 8 years, and late onset of the disease in children above these ages has poorer results compared with onset at lower ages [11]. Current literature suggests that between 30% and 50% of children affected by LCPD will experience hip symptoms in adulthood [12,13]. Previous studies have documented some ethnic and geographical disparities in the incidence of LCPD [14].

**Figure 1:**
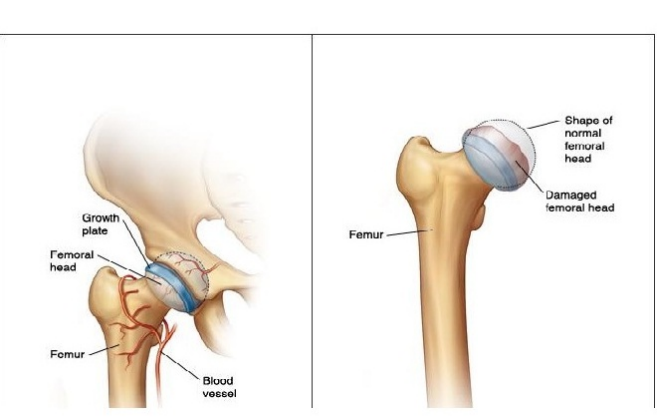
In the normal hip joint, the femoral head is smooth and round (left). In Perthes disease, the femoral head is damaged and loses its normal shape (right).

The formation and progression of LCPD begins with the interruption of femoral head blood supply, which consequently results in changes in the femoral head, metaphysis, growth plate, and acetabulum (Figure 1). Subluxation and lateral displacement of the femoral head out of the acetabulum are among the first signs of the condition [15]. The femoral epiphysis is sensitive to deformation by loading. Lateral migration leads to deformation of the femoral head owing to presence on the edge of the acetabulum and uneven transfer of loading force [16]. Current LCPD treatment focuses on mechanical protection of the femoral head to prevent future hip deformity and degeneration [17], which maintains the plastic epiphysis in the acetabulum and can be done either by noninvasive or surgical techniques [18-20]. Clinicians use the concept of “at risk joint” as the conclusive criterion for the prognosis and treatment options of LCPD [21]. Additionally, imaging methods are used in patient assessment, which provide beneficial information and enable physicians to choose the best case-based strategy for disease management [22,23].

In general, LCPD, which is a childhood hip disorder and is related to interruption of blood supply, progresses over a rage of stages, including necrosis/initial, fragmentation, reossification/healing, and residual [7,24]. Follow-up studies indicate that up to 70% of patients will experience substantial hip pain and dysfunction caused by the disease until adulthood [25,26]. However, the majority of patients have a benign long-term prognosis and need minimal treatment [26]. Treatment of patients focuses primarily on maintaining the femoral head within the acetabulum during the remodeling period [27]. Many studies have been published regarding the treatment options for LCPD; however, the specific therapies are still controversial owing to a poor understanding of its etiology [28]. Treatment options vary from doing nothing to undergoing nonoperative or operative treatments, which have been reported to preserve containment. Nowadays, containment, which can be done with surgical and nonsurgical methods, is suggested as a means for directing the remodeling of the softened femoral head [29-31].

Current treatments for LCPD are largely focused on the early containment of the vulnerable femoral head in the acetabulum to keep the spherical femoral head and congruent joint during the repair period [32,33]. Nonoperative containment options, such as motion therapy, weight relief, and abduction splints, are more appropriate for younger patients, while surgical options are more suggested for older children with more severe LCPD [34]. In the past years, different surgical methods have been developed for treating LCPD, which were claimed to be more appropriate options than nonsurgical treatments for more severe cases of the disease and older patients [1,35,36]. Choosing the best treatment option for the management of LCPD depends on various factors, such as the physician’s own preferences, the patient’s age and disease stage, and the psychosocial status of the patient and family [37-42]. Since various surgical techniques have been proposed for the hips in noncontainable LCPD, the aim of this study was to review the various surgical treatments in LCPD to provide a guide for clinical applications. Some reviews have been published on LCPD management, but each of them has a specific focus. For example, some studies categorized the treatment options as conservative and surgical treatments, with a brief description of each, but in this article, we aimed to review the surgical treatments for LCPD in detail, which differentiates this review from other published studies.

## Methods

### Databases and Search Strategy

We conducted an overview of the English-language literature involving various surgical treatments for LCPD. The electronic databases PubMed, MEDLINE, Web of Science, Embase, and Ovid were searched from October 8 to 14, 2020, for reports on the outcomes of surgical techniques in patients with LCPD. The search was updated on February 4 to 6, 2021. All published studies from January 01, 2000, to the search date were assessed for possible inclusion in this study.

**Figure 2:**
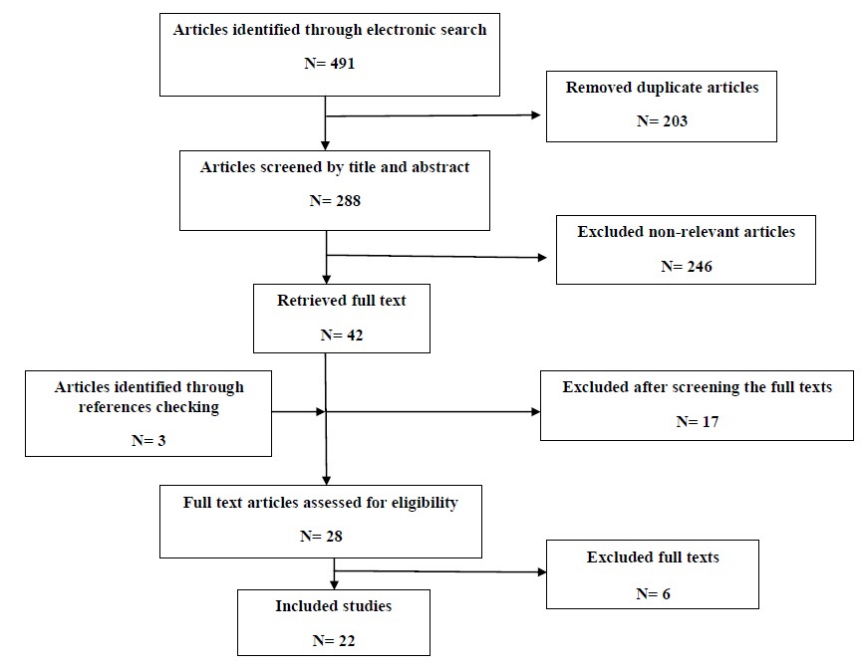
Literature search and review flowchart for the selection of primary studies.

Reference lists of published papers were then hand searched in an attempt to identify further studies (Figure 2). The following keywords were used: Legg-Calvé-Perthes disease, pediatric orthopedic diseases, Perthes disease treatment, avascular necrosis of the hip, osteonecrosis of the femoral head, surgical treatment, osteotomy, hip, and treatment outcome. The search terms were then entered into Google Scholar to ensure that articles were not missed.

### Inclusion Criteria

Studies written in English that reported various aspects of surgical treatments for LCPD and achieved enough quality scores were included in this study.

### Exclusion Criteria

Papers were excluded if they were case reports or had a patient cohort; were not written in English; lacked documentation; and were nonhuman studies, narrative reviews, studies without clinical outcomes data, systematic reviews that did not pool data or perform a meta-analysis, or technique articles without outcomes. We obtained full manuscripts for those studies that met the inclusion criteria.

### Study Selection

Full texts or abstracts of all studies identified during the advanced search were extracted. After excluding duplicates, we investigated the remaining articles by reviewing the titles, abstracts, and full texts. We also reviewed the findings of the articles to prevent reprint bias. Two independent researchers (AM and MNB) selected studies based on the inclusion criteria. Screening was performed after restriction of the search strategy and exclusion of duplicates. Irrelevant studies were removed during the investigation of titles, abstracts, and full texts. The agreement between the selection results of the researchers was assessed based on kappa statistics suggested in the Landis & Koch guidelines [43]. The agreement was considered as slight (kappa 0-0.20), fair (kappa 0.21-0.40), moderate (kappa 0.41-0.60), substantial (kappa 0.61-0.80), and perfect (kappa >0.80). We also reviewed the findings of the articles to prevent reprint bias. Then, the quality of the selected articles was assessed using standard scales.

### Quality Assessment

The quality of primary studies was assessed using appropriate standard checklists. We used the Newcastle-Ottawa Quality Assessment Scale and Jadad Scale for quality assessment of primary studies based on the type of study. Additionally, because the aim of this study was not to combine the results of primary studies using meta-analysis, the effect size was not estimated for the outcome. Therefore, evaluation of heterogeneity was not possible using statistical methods, and results were presented in a purely descriptive form based on the planned design for study.

### Data Extraction and Analysis

All required data, such as authors, publication date, study location, sample size, and treatment technique, were extracted from the included studies using a researcher-made form. The review flowchart is presented in Figure 2.

## Results

### Overview

The characteristics of the included studies [1,9,10,32,44-61] are reported in Multimedia Appendix 1.

### Etiology and Clinical Manifestations

One important predisposing factor for this disease is race, with the East Asian race being affected the least and the White race being affected the most. Additionally, latitude has an influence on susceptibility to Perthes disease [62]. Overall, the reported incidence rate is between 0.2 and 19.1 per 100,000 people [55]. Clinical onset tends to be between 4 and 8 years of age [63]. It has been reported that the incidence increases with the increase in latitude. On the other hand, genetics, repetitive trauma, abnormalities of the blood supply, and coagulation disorders are well-described causative factors [64]. The incidence is the lowest in equatorial regions and increases toward Northern Europe. The incidence is the highest in Whites and the lowest in African Americans [65]. It has been reported that a correlation might exist between acetabular retroversion and Perthes disease. However, the correlation of cause and effect is not known [66]. It has been demonstrated that circulating leptin is higher than normal in patients with LCPD [7]. Therefore, it can be concluded that obesity can play an important role in the initiation of Perthes disease [55].

The prevalence of Perthes disease in boys is five times more than in girls, and 10% to 15% of patients are affected bilaterally; however, bilateral cases are more common in girls [63]. Study findings are conflicting with respect to gender differences in prognosis. Physeal closure in girls occurs earlier, leaving less time for femoral head remodeling [67]. However, no difference between the genders has been detected in final radiographic results. The first presenting complaint is limping, and the second common complaint is pain, which occurs mostly in the anterior hip and medial thigh [55]. The general consensus is that Perthes disease results from the uncoupling of bone metabolism with increased resorption and delayed formation; however, the exact etiology remains unknown. Previous literature states that as patients with LCPD tend to have delayed bone age (on average, 2 years in girls and 1 year in boys), their femoral head ossific nuclei are smaller than those in children of similar age [34]. This makes the cartilaginous component of their epiphysis larger, and the traversing blood vessels are more vulnerable to mechanical compression [68].

### Imaging

Simple radiographs remain the most useful imaging modality, which can be used for the initial diagnosis of LCPD and subsequent follow-up. The size and shape of the femoral head are of importance in this approach [55,63] Characteristic changes usually occur after a radiographically silent period in the first 3 to 6 months of the disease. A relatively thickened cartilage may widen the medial joint space. The involved hip has a smaller ossific nucleus, often with increased radiodensity. An increase in the joint space has been shown to be correlated with enlargement of the femoral head [69]. Prognostic radiographic signs rarely appear until Perthes disease is established, and this usually takes over 6 months after disease onset. Other techniques, such as magnetic resonance imaging and pneumoarthrography, can provide more comprehensive information regarding the stage of the disease [70].

Arthrography as an adjunct to standard radiography aids in the assessment of the range of motion and ability to contain the head in the acetabulum. After general anesthesia and strict sterile preparation, contrast is injected with fluoroscopic guidance to examine the features [71]. Sonography has been reported to detect hip effusion early in the disease when radiographs are undiagnostic [72]. Three-dimensional computed tomography can show early bone collapse, so it can be useful in visualizing complex head deformity, but the benefit of the information gained rarely justifies the radiation dose required [63]. Magnetic resonance imaging details the extent of bony infarction and the anatomy of the cartilaginous head and labrum, which can be useful early in the disease’s course to differentiate it from other conditions that cause osteonecrosis [73,74]. In bone scanning, there is a strong correlation between the size of the uptake defect on the femoral head and prognosis. The indication is limited to patients who are suspected of being affected by LCPD, which further serves as a prognosticator as well [75]. A previous study showed that there is a significant correlation between hip deformity and labral and cartilage abnormalities of the hip on magnetic resonance imaging, and the main predisposing factors were loss of sphericity of the head and a decline in femoral head-neck offset [76].

### Surgical Treatment Options

The treatment of Perthes disease depends on the age and stage of presentation. Simple observation is needed in children aged 2 to 3 years. The optimal treatment technique for LCPD and its prognosis are still not fully understood. In the past 20 years, some authors have tried to standardize the treatment principles for Perthes hip. Extent of femoral head involvement (lateral pillar classification or Catterall classification) and age at diagnosis are the most common classifications used to assess the outcomes following treatment [55]. In a large prospective review by Wiig et al [32], with medium-term follow-up, it was suggested that children aged 6 years or older, with more than 50% femoral head involvement (Catterall), had a better result if treated with surgery.

#### Arthrodiastasis

Arthrodiastasis is a relatively novel treatment method for LCPD, which uses an external fixator. Arthrodiastasis was initially used to describe a technique involving articulated distraction of the hip joint that was developed by surgeons in Verona, Italy, and has been used since 1979 [77]. It has been considered as an alternative treatment for LCPD beyond conventional surgical methods. This method was conceived as a conservative technique of restoring joint function, based on awareness that under certain conditions, regeneration and repair of damaged articular cartilage can occur, at least to some extent [78]. It is considered to be useful because it maintains the mobility of the hip joint and secures space for the femoral head in the joint while minimizing physical pressure and preserving synovial fluid circulation. Kim et al [79] reported that arthrodiastasis using an external fixator can be a relatively promising surgical procedure for the treatment of late-onset LCPD. Additionally, a systematic review by Ibrahim et al [80] investigated relevant literature to assess the efficacy of the use of arthrodiastasis in the management of Perthes disease and showed a significant increase in the postoperative range of motion compared with the preoperative range of motion. Final Stulberg classification was ascertained, and the majority of patients were in stages 2 and 3. Complications were also assessed, with the majority of them being superficial pin tract infections. They concluded that arthrodiastasis is a valid treatment option for Perthes disease; however, more studies need to be performed showing comparative data of arthrodiastasis versus other containment procedures. Arthrodiastasis of the hip joint with soft tissue release is considered as a surgical technique when other treatment options are contraindicated. This method also improves the range of motion, decreases superior and lateral subluxation, and provides better radiographic sphericity of the femoral head. Treatment with distraction may be performed even for stiff hips and hips with deformity [81]. Volpon [17] performed a prospective controlled trial to compare innominate osteotomy and arthrodistraction and concluded that despite similar final radiological outcomes, arthrodistraction was associated with higher morbidity; therefore, hip distraction is not recommended as the primary treatment in the early stages of LCPD.

#### Salter Osteotomy

Salter osteotomy, as a method for surgical containment in LCPD, was first introduced in 1962. This technique redirects the acetabulum as well as improves anterolateral femoral head coverage. Salter presented the concept of innominate osteotomy as a containment technique to avoid femoral osteotomy consequences [82]. Salter felt that acetabular rotation would also provide better containment than varus osteotomy of the femur; however, studies have shown little difference in the radiographic or functional results with either of these two techniques. The common indications for salter osteotomy are similar to other forms of containment [39]. Some of these indications include onset age over 6 years, more than 50% of the femoral head affected, and hip subluxation in the weight-bearing position. This operative method has been reported to produce better long-term outcomes than nonoperative techniques with regard to Stulberg classification [34]. Several studies [83,84] have compared Salter osteotomy and femoral varus osteotomy. Previous studies reported similar outcomes with respect to femoral head sphericity, but have shown increased femoral head coverage by the center-edge angle after Salter osteotomy [85]. Use of this technique can displace the acetabulum 1 cm medially and distally, thereby reducing the biomechanical stress over the hip joint and improving the generally associated leg length discrepancy [86]. It should be noted that radiographic assessment as well as cautious clinical examination is necessary before surgery. Some of the Salter osteotomy prerequisites include full range of hip motion preoperatively, especially abduction, and reasonable joint congruency [10].

The main benefit of Salter or innominate osteotomy is its effect on femoral head remodeling during remaining growth. This osteotomy alone is commonly indicated for younger children with recent clinical onset and no femoral head deformity or subluxation [39]. However, Salter osteotomy alone may not provide sufficient head coverage in all situations, especially in children older than 9 years. Thus, the combination of Salter and femoral varus osteotomies has been performed recently to manage a larger and deformed femoral head [40,87]. A previous study stated that the combined method of surgery may change the otherwise “poor” hip into a “fair” hip and improve the natural history in children with higher age [87]. The other advantages of the combined method include a reduction in the effect of increased intra-articular pressure from innominate osteotomy and compensation of the shortening from femoral osteotomy [10].

#### Femoral Varus Osteotomy

Femoral varus osteotomy has become one of the most popular surgical techniques for Perthes disease, since the first report by Axer in 1965 [38,88]. The aim of this method is to center the femoral head deeply within the acetabulum and allow correction of the flexion or rotational deformity simultaneously [10]. The prerequisites for this technique are good range of motion, hip congruency, and ability to contain the femoral head in abduction. This surgery is suggested in the early stage of fragmentation, when favorable biological and biomechanical effects may be anticipated. Many studies reported that femoral varus osteotomy yields good long-term outcomes [37,38]. The reported number of hips treated operatively rose more sharply during the last decade in research from Europe and North America. With regard to the type of surgical treatment, femoral osteotomy was reported more frequently than pelvic osteotomy worldwide; however, pelvic osteotomy is comparably more common in North America, Australia, and South America, whereas femoral osteotomy is more frequently performed in Europe, Asia, and Africa [1].

The main goal of surgical methods is to contain the femoral head within the acetabulum in order to avoid femoral head deformation and subsequent premature hip osteoarthritis. This aim is achievable by the use of femoral varus osteotomy, innominate osteotomy, and other forms of pelvic osteotomies. Operative treatments can roughly be categorized as femoral, pelvic, and combined procedures. A comprehensive review by Braito et al [1] stated that femoral osteotomies were reportedly more frequent than pelvic osteotomies in the screened literature. They concluded that femoral osteotomies were tendentially preferred in Europe. Saran et al [24] showed that children older than 6 years benefit more from varus osteotomy compared with nonoperative treatments. Generally, femoral varus osteotomy allows realignment and identification of the best fit position of the hip, while restoring joint congruity and decreasing femoroacetabular impingement.

### Combined Treatments

Any pelvic osteotomy can be combined with a proximal femoral osteotomy, especially if the femoral head cannot be contained by a pelvic or proximal femoral varus osteotomy alone [39]. The combined Salter and proximal femoral varus osteotomy for LCPD has been performed more recently [89,90]. These combined procedures are usually used for patients with an older age at clinical onset, those with deformed femoral heads, or those in whom osteotomy alone cannot provide adequate containment [39]. Javid and Wedge [87] used combined osteotomies in 20 older patients with LCPD and reported that outcomes improved with the combined osteotomies at skeletal maturity when compared to the natural history of untreated hips. Vukasinovic et al [90] investigated patients treated with combined Salter and proximal femoral shortening osteotomy. They showed a better center-edge angle in these patients. Their results were similar to those reported by other researchers [83,84].

#### Chiari Osteotomy

Chiari osteotomy is a popular salvage procedure for children with insufficient femoral head coverage [10]. One of the advantages of this method is the reduction of joint loading by medialization of the hip, which was considered an important factor for improving hip congruency and femoral head remodeling [91]. This technique has been recommended for severe cases of Perthes disease. Medial displacement or Chiari osteotomy is one of the categories of pelvic osteotomies. The most performed methods in Perthes disease are acetabular rotational osteotomies, especially Salter osteotomy. The Chiari medial displacement osteotomy procedure is usually used for salvage of a deformed femoral head [39].

#### Triple Innominate Osteotomy

Triple innominate osteotomy is another option for achieving containment in LCPD. Femoral varus osteotomy and Salter osteotomy are the most common techniques for surgical containment; however, the degree of femoral varus osteotomy required to contain the femoral head may further shorten the limb and cause prolonged limp, particularly in older children. On the other hand, use of Salter osteotomy may not provide enough acetabular rotation to cover the femoral head in severe cases, potentially leading to iatrogenic hinge abduction [92]. Because of certain practical limitations with these two procedures, advanced containment methods, such as triple innominate osteotomy, have been developed for more severe cases [40,93]. Some studies have reported that older age and extensive femoral head involvement were risk factors for unsatisfactory outcomes. A previous study showed that patients older than 10 years at onset had poor results regardless of surgical treatment [94]. Triple innominate osteotomy is anticipated to show better femoral head containment than can be achieved with Salter osteotomy alone and to avoid the leg length discrepancy associated with femoral varus osteotomy. Finally, this is one of the most efficient methods for femoral head containment in all conditions. However, over coverage can result in pincer impingement. For the prevention of pincer impingement, correction beyond 44 degrees of the enter-edge angle is not recommended [95].

### Other Treatment Options

Lateral shelf acetabuloplasty is considered for severe Perthes disease when redirection osteotomy is thought to be insufficient to produce optimal coverage of the extruded femoral head. An intraoperative dynamic arthrograph is useful for further confirmation. In severe cases, a laterally displaced and enlarged femoral head will preclude normal motion of the hip. Previous reports have shown that shelf acetabuloplasty is a safe and effective method for managing cases with aspherical congruency or incongruency with hinge abduction [42,96]. When an arthrograph indicates femoral head deformity with unstable movement and hinge abduction, but stability in adduction and flexion, valgus and extension osteotomy can be an effective method for unloading the deformed epiphyseal segment and alleviating femoroacetabular impingement. The implication of femoral valgus extension osteotomy depends on redirection of the more congruent and round anteromedial part of the femoral head to the neutral position of weight bearing. This sagittal and rotational correction may improve gait and hip motion, decrease pain, and improve femoral head shape [97,98].

Transtrochanteric rotational osteotomy is considered a new technique for patients with onset of LCPD after 9 years of age. It is an effective method to treat late-onset Perthes disease in affected hips. In addition, the amount of head involvement and the lateral pillar influence surgical results [44]. Recent techniques are focused on reshaping the femoral head to match with the acetabulum and reduce impingement, as well as restoring the normal cartilage in the head weight-bearing zone [45]. Total hip arthroplasty is a salvage method for complications and subsequent osteoarthritis. Cementless total hip arthroplasty showed a 90% survival rate in an 8-year follow-up. However, despite promising outcomes, nerve injury and intraoperative fracture are usual; therefore, care should be taken to avoid excessive limb lengthening [46].

## Discussion

In this study, we reviewed the various surgical treatments for LCPD to provide a guide for clinical applications. Based on the results of the reviewed studies, there are three main factors that influence the treatment outcomes in patients with Perthes disease. These factors include onset age, femoral head involvement severity, and treatment method. For patients aged over 8 years, the prognosis is often poor, but advanced or salvage procedures still provide the benefit of improved femoral head coverage; therefore, they benefit from surgical intervention. For children aged less than 6 years, the prognosis is generally good. For children aged between 6 and 8 years, the prognosis is variable, and it is required to closely observe for the signs of “head at risk,” which indicate the need for operation. Once any such signs are observed, dynamic arthrography under anesthesia is valuable before deciding the appropriate treatment approach.

## Appendix

Multimedia Appendix 1 The characteristics of the included studies.

## Abbreviations

LCPD: Legg-Calvé-Perthes disease
