# Supplementary material for: Surgical Treatments for Legg-Calvé-Perthes Disease: Comprehensive Review
Source: Interact J Med Res. 2021 May 3;10(2):e27075. doi: 10.2196/27075 (PMC8129878; doi:10.2196/27075)
Supplement: Multimedia Appendix 1 [file ijmr_v10i2e27075_app1.doc]

**Multimedia Appendix 1. The characteristics of the included studies.**

| **Author(s) [Ref.]** | **Year** | **Location** | **Sample size** | **Method** | **Intervention** |
| --- | --- | --- | --- | --- | --- |
| Braito et al. [1] | 2020 |  | 123 primary studies | Review | Various methods |
| Erkus et al. [43] | 2020 | Turkey | 47 patients | Cross-sectional study | Various methods |
| Ramachandran and Reed [44] | 2020 | UK | 59 references | Review | Various methods |
| Kamegaya et al. [45] | 2018 | Japan | 77 patients with average 9.5 years follow-up | Retrospective study | Combined Procedures |
| Mosow et al. [46] | 2017 | Germany | 52 patients | Retrospective study | Pelvic and Femoral Osteotomies |
| Park et al. [47] | 2017 | Korea | 29 patients | Follow up study | Modified Salter Innominate Osteotomy |
| Stepanovich et al. [48] | 2017 | USA | 54 patients | Retrospective review study | Triple Innominate Osteotomy |
| Shohat et al. [49] | 2016 | Israil | 35 patients | Long-term follow-up study | Varus Derotational Osteotomy |
| Li and Xu [50] | 2016 | China | 51 patients | Long-term follow-up study | Lateral shelf acetabuloplasty |
| Carsi et al. [51] | 2015 | UK | 44 patients | Retrospective series study | Shelf acetabuloplasty |
| Lim and Shim. [52] | 2015 | Korea | 12 patients | Retrospective study | Combined SA with Femoral Varus Osteotomy |
| Bulut et al. [53] | 2014 | Turkey | 16 patients | Long-term follow-up study | Salter pelvic osteotomy |
| Mazloumi et al. [54] | 2014 | Iran | 54 references | Concept review | Various methods |
| Yoo et al. [55] | 2013 | Korea | 31 patients | Retrospective case series | Valgus femoral osteotomy |
| Terjesen et al. [56] | 2012 | Norway | 70 patients | Nationwide prospective study | Varus femoral osteotomy |
| Wu et al. [10] | 2012 | - | 44 references | Review | Various methods |
| Hosny et al. [57] | 2011 | Egypt | 33 patients | Long-term follow-up study | Arthrodiastasis |
| Glard et al. [58] | 2009 | France | 58 patients in 2 groups | Retrospective comparison of two matched groups of  Patients (operative and non-operative) | Varus |
| Castaneda et al. [59] | 2008 | USA | 121 patients | Retrospective comparison of two groups of  Patients (operative and non-operative) | Varus |
| Wiig et al. [32] | 2008 | Norway | Norway | Prospective follow-up study | Varus |
| Rowe et al. [60] | 2005 | Korea | 68 patients | Longitudinal study | Brace, varus |
| Herring et al. [9] | 2004 | USA | 345 | controlled, long-term multicenter study | Various methods |
